# The Population Size and Distribution of Diurnal Large Wild Mammals in the Southern Great Rift Valley, Ethiopia

**DOI:** 10.1155/2022/3050710

**Published:** 2022-02-27

**Authors:** Yacob Kassa, Wondimagegnehu Tekalign

**Affiliations:** Wolaita Sodo University, Department of Biology, College of Natural Sciences, P.O. Box 138, Sodo, Ethiopia

## Abstract

The study was carried out to assess the population size and distribution of diurnal large wild mammals in the southern Great Rift Valley, Ethiopia. The study area was stratified into four habitat types: riverine forest, ground-water forest, grassland, and bushland. Samples of animals were surveyed through the transect method. The total number of individuals belonging to the 15 species observed was 1681 and 1245 during the wet and dry seasons, respectively. Burchell's zebra (*Equus burchellii*), Anubis baboon (*Papio anubis*), Vervet monkey (*Chlorocebus pygerythrus*), and Grant's gazelle (*Nanger granti*) were the most abundant species, while Abyssinian hare (*Lepus habessinicus*) and Bush duiker (*Sylvicapra grimmia*) were the least abundant species. The highest number of species has been supported by the bushland habitat, followed by open grassland, riverine forest, and ground-water forest in both seasons. Despite the park being home to various types of mammalian species, there is a need for conservation actions by the park management and other concerned bodies for the survival of those species in the area.

## 1. Introduction

Diurnal large mammals have a special role in maintaining important ecological functions of terrestrial ecosystems and are good indicators of habitat value since they contribute to the conservation efforts of other species [1, 2]. In particular, large predators often shape the population size, distribution, and behavioral activities of prey populations [3], and large herbivores act as ecological engineers by changing the structure and species composition of the vegetation [4]. Beyond the direct species interaction, mammalian species manipulate the whole ecosystem through cascading trophic effects [3, 5].

The high diversity of large mammals is a natural feature of the African tropical savanna biomes and the present distribution of such species within the topographically diverse Rift Valley region of the East African savanna [6].

Understanding the distribution, abundance, and habitat requirements of mammalian species is basic to establishing a baseline for their long-term monitoring at a particular site. Even though high mammalian species diversity is present in Ethiopia, its mammalian species have declined in recent years, and there is little information about the mammalian resources [7]. Nech Sar National Park is one of the protected areas in southern Ethiopia which is thought to be home to a variety of wildlife including large mammals [8]. Nevertheless, very little published information exists about large mammalian species.

Therefore, the present study aimed to assess the population size and distribution of diurnal large wild mammals along systematically laid transects in the study area. This study will contribute to the filling of some information gaps and provide current information on the large mammals for the strong management actions in the national park area.

## 2. Methods and Materials

### 2.1. The Study Area

**Figure 1:**
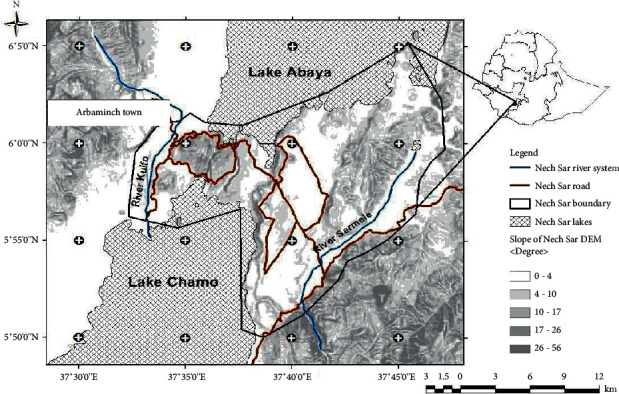
Location map of Nech Sar National Park [26].

Nech Sar National Park is one of the national parks of southern Ethiopia and is home to great habitat/species diversity (Figure 1). The park was established in 1974 in the scenic part of the Rift Valley floor between the Lakes Abaya and Chamo, adjacent to Arbaminch town. It comprises 514 km^2^ of which 85% island-covered and the remaining 15% is water [9]. It is located between the latitudes of 5051′N to 6050′N and the longitudes of 37032′E to 37048′E with elevations ranging between 1108 m and 1650 m asl. The temperature ranges from 12.2°C to 34.3°C.

The lakes and lakeshore areas are an interesting component of the great biodiversity of the park ecosystem. Lake Chamo supports a high density of very large Nile crocodiles with a particular concentration of them at the beach known as the Crocodile Market, the largest hippo population in Ethiopia, and abundant fish including Nile perch and water-related birds. The NechSar plains are the dominant feature of the national park and the main source of food for grazing animals.

### 2.2. Methods

The study area was stratified into four main study units or “census zones” based on the main vegetation types, and data were collected from these census zones, such as riverine forest (30.1 km^2^), ground-water forest (37.4 km^2^), grassland (270 km^2^), and bushland (80.87 km^2^) census zones through line-transect survey following the work of Sutherland [10].

**Figure 2:**
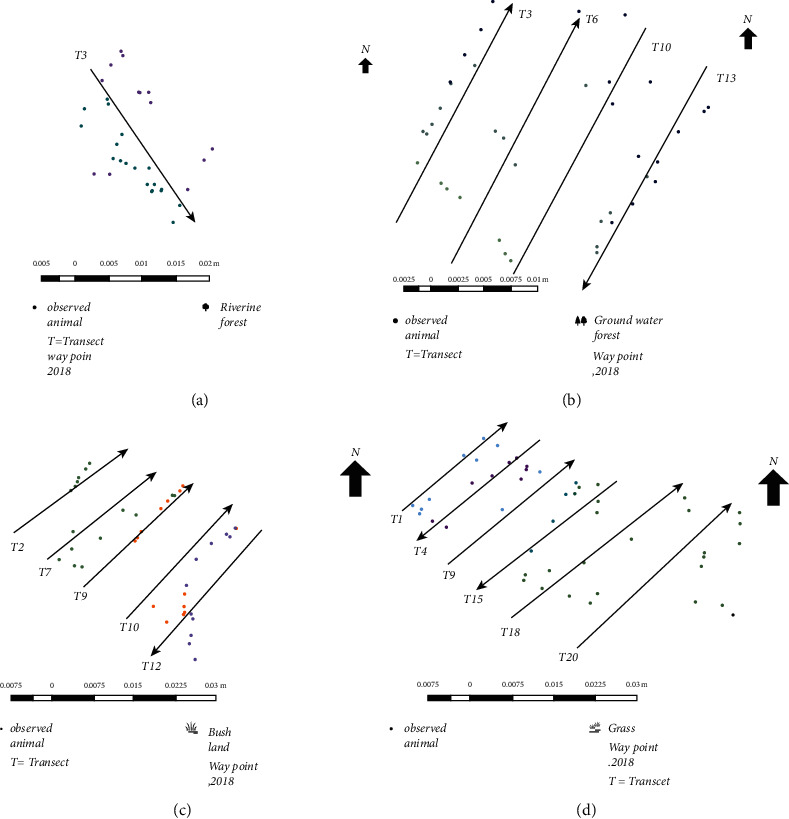
Map of sampled transect in (a) riverine forest habitat; (b) groundwater forest habitat; (c) bushland habitat; and (d) grassland habitat.

A total of 16 transect lines was established; six for grassland, five for bushland, four for the ground-water forest, and the remaining one for the riverine forest. The sampling transects selected from each census zone represented about 25–30% of each census zone [11] (Table 1). The study was conducted during both the dry (December 2017–February 2018) and wet (March–May 2018) seasons. A survey was conducted using GPS and binoculars in each randomly selected block along the selected transects. The transect width varied from 100 m to 500 m. The variation was determined based on the type of vegetation cover of each of the census zones. The length of transects also varied from 3.5 to 5 km and was determined based on the type of ecological units (Figure 2).

The survey was conducted on foot along the established transects observing the prevailing mammals on each transect's left and right sides. Two observers were involved in collecting data from the left and right sides of each line transect. Both were assigned to the left or right side of the transect line and scanned the route with the spotlight. Accordingly, all transects were visited bimonthly during the data collection periods of both the wet and dry seasons. To enhance sampling effort, in a single visit, each transect was walked twice: early in the morning during 06:00–10:00 am and late in the afternoon during 03:00–06:00 pm when the wild animals were more active.

Whenever an individual or group of mammals were observed, group size, sighting distance (defined as the distance from each line transect to the geometric center of the group or individual), and sighting angle between the transect line and individual or group were recorded on the datasheet. For the direct sighting, the naked eye and Bushnell laser rangefinder binoculars were used. The starting and ending points of each transect were fed into a Garmin GPS unit and used for navigation during data collection. The perpendicular distance from the transect line to the animal was calculated. The same transect was used to carry out a census during the investigation period. When the animals were observed, vegetation or other obstacles might have hindered clear visibility. Then, the observer silently approached them by leaving the transect route; however, the sighting distance was measured from the centerline to the animals. Double recording of the same individual or group in a single visit was avoided to the extent possible using easily recognizable features such as cluster size, harem composition, and distinct individuals with body deformities such as cut tail and ear of the individual or group size and composition.

For the purpose of this study, large mammals were defined as all mammals (focus on herbivores and primates) with an average weight of ≥2 kg and were detected with direct observation. Identification and recording of the numbers of large mammalian species were made through direct observation with the naked eye and/or aided with binoculars (7 × 50 mm imaging). Kingdon and Largen's [12] field guidebook was used for the identification of mammals. Field identification of diurnal mammalian species was done based on visible morphological characters. To have clear pictures of each mammal, observer noise was minimized as much as possible by walking quietly and gently at a constant speed along each transect, against the direction of the wind, to minimize disturbances of mammalian species. The location points of each mammalian species, whether group or individual in the field at each habitat type, were also identified and recorded using GPS to map their distribution.

### 2.3. Data Analysis

The data collected in the present study were analyzed by the use of SPSS version 20. The chi-square test was also used to compare the seasonal variations in species composition and abundance of individuals among habitats at 0.05 levels of significance. The abundance of mammalian species in each of the habitats was calculated as follows: abundance = the total number of individuals of a species/sampled habitats.

## 3. Results

During the present investigation, a total of 15 diurnal large mammalian species (herbivores and primates) were identified and recorded in the Nech Sar National Park in both the dry and wet seasons. In the survey, all of these species were recorded within the randomly selected sampling habitats of the four major habitat types. The numbers of mammals recorded in the four habitat types of the park were as follows: in the grassland habitat, 568 and 351 were followed by bushland with 506 and 326, ground-water forest with 341 and 354, and riverine forest habitat with 266 and 214, during both wet and dry seasons, respectively (Tables 2 and 3). Seasonal variations in the abundance of individuals between wet and dry seasons in groundwater forest habitat (*χ*2 = 43, df = 1, *p* < 0.05), riverine forest (*χ*2 = 52.10, df = 1, *p* < 0.05), bushland (*χ*2 = 38, df = 2, *p* < 0.05), and grassland habitat (*χ*2 = 56, df = 2, *p* < 0.05)were significantly different.

There was no difference in the total number of individual species recorded during both seasons. The total number of mammals belonging to the different species recorded during the wet season survey was 1681 (57.45%), while in the dry season it was 1245 (42.55%). There was a marked difference in the total number of mammals recorded during the dry and wet seasons (*χ*2 = 127.309, df = 1, *p* < 0.05).

The relative abundance of different individual species of the study area varied from 0.36 to 29.80% in the wet season and from 0.32 to 27.55% in the dry season. The most abundant species in the sampled area during the wet and dry seasons were Burchell's zebra (*n* = 501 and *n* = 343), Anubis baboon (*n* = 412 and *n* = 332), Vervet monkey (*n* = 157 and *n* = 116), Grant's gazelle (*n* = 151 and *n* = 94), and Colobus monkey (*n* = 124 and *n* = 135), respectively. This was followed by warthog (*n* = 63 and *n* = 41), hippopotamus (*n* = 53 and *n* = 28), and greater kudu (*n* = 48 and *n* = 28), respectively. Abyssinian hare (*n* = 15 and *n* = 9) and Bush duiker (*n* = 6 and *n* = 4) were the least abundant species in the study area in both the wet and dry seasons, respectively (Table 3).

The relative abundance of individual species in the groundwater forest habitat varied between 0–50.73% and 0–46.61% during the wet and dry seasons, respectively. In the riverine forest habitat, it was between 0 and 52.63% and between 0 and 51.87% in the wet and dry season, respectively. For the bushland habitat, it was between 1.19–37.75% and 0.31–42.94% in the wet and dry seasons, respectively. In the grassland habitat, the relative abundance was between 0 and 54.58% and 0–57.83 percent during the wet and dry seasons, respectively (Table 3).

## 4. Discussion

Large mammals' distribution and abundance in the present study were highly associated with habitat types. In this study, the bushland habitat supported the highest number of mammalian species followed by open grassland, riverine forest, and groundwater forest in both wet and dry seasons. A similar result was obtained by Lemma and Tekalign [13] in the Humbo Community Based Forest, in which the highest numbers of mammals were found in the bushland area, followed by the open grassland, while the riverine forest supported the least number of mammalian species. However, the study of Chane and Yirga [14] in Borena-Sayint National Park indicated that woodland habitat has supported the highest number of mammalian species, followed by the riverine forest and open grassland habitat, respectively. The possible reasons for this distribution of large mammalian species might be due to the presence of food, water, and stability of the area from human disturbances. According to Tolcha et al. [15], the availability of quality forage and other resources determines the habitat preference and association of ungulates. Besides, the effects of different predator abundances not captured in the present study might also be possible drivers affecting the habitat selection by the antelope species that have been shown to avoid certain habitat types due to an increased risk of predation [16, 17]. Large mammals in this study area had no consistent distribution among the habitat types. Therefore, their abundance significantly varies among habitats between seasons. Balakrishnan and Easa [18] also described that water and pasture conditions or the combinations of both are the major factors determining the distribution of wildlife populations in their natural habitats.

The five large mammalian species, the Burchell's zebra, Anubis baboon, Vervet monkey, Grant's gazelle, and Colobus monkey were the most abundant in both wet and dry seasons. Burchell's zebra and Grant's gazelle were favored in the bushland and open grassland habitats. However, Anubis baboon was found in all four habitats, and Vervet monkey and Colobus monkey were totally absent from the open and bushy grassland. This might be attributed to the feeding behavior that it is adapted to feed on a variety of food items. Kingdon and Largen [12] described that primates commonly need forested areas with tall trees. A large number of Colobus monkey individuals were recorded in the present study from the habitats where that existed during both the wet and dry seasons. Guenther's dik-dik, Bushpig, Abyssinian hare, and Bush duiker were the least abundant during this study. The probable reason for this could be the growth of herbaceous and ground vegetation, which provides dense concealment for mammalian species, which makes detection problematic [19, 20].

Burchell's zebra or plains zebra was closely associated with the availability of water and edible grasses. The present study showed that plains zebra live in areas where few other plain ungulates and livestock also occur. The number of individuals of lesser kudu that were recorded in the habitats of open grassland and bushland during both seasons is comparable with the individuals recorded by C. Stuart and T. Stuart [21]. These animals have frequently occurred in the open woodland, less frequent in the wooded grasslands, and the least observed in shrublands and open grassland areas. These animals were hardly observed in dense forests, riverine forests, and waterlogged areas. The habitat choice of animals in the study area is probably a consequence of competition and predation. The number of individuals of large mammals recorded during the wet season surpassed the number recorded during the dry season. In the open grassland and groundwater forest area was the common habitat for livestock and human encroachment, which was thought to be an ideal habitat for Burchell's zebra and Anubis baboon species.

Gundogdu [22] showed that livestock and human encroachments often reduce the foraging opportunities of wild mammals, which in turn reduce the mammal's opportunities of being sighted. From the households in the nearby villages of the Nech Sar National Park, a total of 7587 heads of cattle and goats were recorded by Doku et al. [23]. Fetene et al. [24] indicated that, within the Nech Sar National Park, between 2005 and 2013, the number of livestock had increased by half. Higher grazing pressure depreciates the scenery and the visibility of the wildlife species of the park [25]. Besides, firewood collection and harvesting of grasses were higher in the dry season, thus likely reducing the sighting of mammals. According to Fetene et al. [24], fuelwood and construction wood gathering are also everyday activities that have a high effect on the wildlife habitats of the park. Fetene et al. [24] also identified that grazing negatively affects and deteriorates the scenery and the wildlife visibility diminishing greatly over time and putting obstacles for the income-generating from ecotourism activities of the park.

Personal observations during the study period indicated that the park was surrounded by agricultural communities from its eastern and western boundaries that might cause the expansion of settlement in and/or around the park. Girma and Stellmacher [26] stated that wildlife species might decline as the level of development in the surrounding natural habitat has increased through the modification of vegetation structure and composition by human settlers.

Therefore, there is a need for intervention by various stakeholders including the adjacent communities, to alleviate any devastating effects on the area and on the existing wildlife resources with special consideration for the large mammalian fauna of the park. The Ethiopian Wildlife Conservation Authority should also design an appropriate management plan to upgrade the current status with all the logistics and personnel recommended as a conservation measure.

## Figures and Tables

**Table 1:** Number, length, width, and percentage of the habitat type covered by utilizing the transects for randomly selected transects.

Census zones	Potential transects	Sampled transects	Length and width of transects (km × km)	Percentage of the habitat type covered
Groundwater forest	13	4	3.5 × 0.1	30.00
Riverine forest	4	1	4.5 × 0.1	25.00
Bushland	16	5	3.5 × 0.3	30.00
Grassland	20	6	5 × 0.5	30.00

**Table 2:** The relative abundance of large mammals during the wet and dry seasons.

Common name	Species name	IUCN red list category	Season			
			Wet		Dry	
			Number of individuals	Relative abundance	Number of individuals	Relative abundance
Burchell's zebra	*Equus burchellii*	NT	501	29.80	343	27.55
Anubis baboon	*Papio anubis*	LC	412	24.51	332	26.67
Vervet monkey	*Chlorocebus pygerythrus*	LC	157	9.34	116	9.32
Grant's gazelle	*Nanger granti*	LC	151	8.98	94	7.55
Colobus monkey	*Colobus guereza*	LC	124	7.38	135	10.84
Warthog	*Phacochoerus africanus*	LC	63	3.75	41	3.29
Hippopotamus	*Hippopotamus amphibious*	VU	53	3.15	28	2.25
Greater kudu	*Tragelaphus strepsiceros*	LC	48	2.86	28	2.25
Lesser kudu	*Tragelaphus imberbis*	NT	36	2.14	25	2.01
Waterbuck	*Kobus ellipsiprymnus*	LC	32	1.90	35	2.81
Bushbuck	*Tragelaphus sylvaticus*	LC	32	1.90	37	2.97
Guenther's dik-dik	*Madoqua guentheri*	LC	26	1.55	6	0.48
Bushpig	*Potamochoerus larvatus*	LC	25	1.49	12	0.96
Abyssinian hare	*Lepus habessinicus*	LCs	15	0.89	9	0.73
Bush duiker	*Sylvicapra grimmia*	LC	6	0.36	4	0.32
Total		1681	100	1245	100	

*Note.* LC, least concern; VU, vulnerable; NT, near threatened.

**Table 3:** Relative abundance of large mammals in four habitat types during the wet and dry season.

Common name	Species name	GWF		RF		BL		GL	
		Wet	Dry	Wet	Dry	Wet	Dry	Wet	Dry
Burchell's zebra	*Equus burchellii*	0.00	0.00	0.00	0.00	37.75	42.94	54.58	57.83
Anubis baboon	*Papio anubis*	50.73	46.61	52.63	51.87	12.65	11.96	6.16	4.84
Vervet monkey	*Chlorocebus pygerythrus*	26.98	24.29	14.66	7.48	5.14	4.29	0.00	0.00
Grant's gazelle	*Nanger granti*	0.00	0.00	0.00	0.00	7.11	7.06	20.25	20.23
Colobus monkey	*Colobus guereza*	15.25	22.88	12.78	25.23	7.50	0.00	0.00	0.00
Warthog	*Phacochoerus africanus*	3.81	2.54	6.02	4.67	4.55	3.99	1.94	2.56
Hippopotamus	*Hippopotamus amphibious*	0.00	0.00	0.00	0.00	6.32	6.13	3.70	2.28
Greater kudu	*Tragelaphus strepsiceros*	0.00	0.00	1.88	1.87	3.16	4.29	4.75	2.85
Lesser kudu	*Tragelaphus imberbis*	0.00	0.85	5.26	6.08	3.56	5.83	0.00	0.00
Water buck	*Kobus ellipsiprymnus*	0.00	0.00	0.00	0.00	2.77	2.77	3.87	4.56
Bushbuck	*Tragelaphus sylvaticus*	0.00	0.00	2.63	0.93	2.96	7.06	1.76	3.43
Bushpig	*Potamochoerus larvatus*	2.35	1.69	3.38	1.87	1.58	0.61	0.00	0.00
Guenther's dik-dik	*Madoqua guentheri*	0.59	0.00	0.76	0.00	2.57	1.23	1.58	0.57
Abyssinian hare	*Lepus habessinicus*	0.29	0.85	0.00	0.00	1.19	1.53	1.41	0.28
Bush duiker	*Sylvicapra girmma*	0.00	0.29	0.00	0.00	1.19	0.31	0.00	0.57

GWF: groundwater forest, RF: riverine forest, BL: bushland, and GL: grassland.

## Data Availability

No data were used to support this study.

## References

[1] Estes J. A., Terborgh J., Brashares J. S. (2011). Trophic downgrading of planet earth. *Science*.

[2] Udy K., Fritsch M., Meyer K. M. (2021). Environmental heterogeneity predicts global species richness patterns better than area. *Global Ecology and Biogeography*.

[3] Berger J., Stacey P., Bellis L., Johnson M. P. (2001). A mammalian predator-prey imbalance: grizzly bear and wolf extinction affect avian neotropical migrants. *Ecological Applications*.

[4] Dinerstein E. (2003). *The Return of the Unicorns*.

[5] Crooks K. R., Soulé M. E. (1999). Mesopredator release and avifaunal extinctions in a fragmented system. *Nature*.

[6] Turpie J. K., Crowe T. M. (1994). Patterns of distribution, diversity and endemism of larger african mammals. *South African Journal of Zoology*.

[7] Girma Z., Mammo Y., Ersado M. (2012). Species composition, distribution and relative abundance of large mammals in and around Wondo genet forest patch, Southern Ethiopia. *Asian Journal of Applied Sciences*.

[8] Vreugdenhil D., Vreugdenhil A. M., Tilahun T., Shimelis A., Tefera Z. (2012). *Gap Analysis of the Protected Areas Oystem of Ethiopia*.

[9] EWCA (Ethiopian Wildlife Conservation Authority) (1999). *General Description of the Nech Sar National Park Produced Information Bulletin*.

[10] Sutherland W. J. (2006). *Ecological Census Techniques: A Handbook*.

[11] Yates F. (1960). *Sampling Methods for Censuses and Surveys*.

[12] Kingdon J., Largen M. (2003). The kingdon field guide to african mammals. *Zoological Journal of the Linnean Society*.

[13] Lemma A., Tekalign W. (2020). Abundance, species diversity, and distribution of diurnalmammals in Humbo community-based forest area, Southern Ethiopia. *International Journal of Zoology*.

[14] Chane M., Yirga S. (2014). Diversity of medium and large-sized mammals in Borena-Sayint National Park, South Wollo, Ethiopia. *Intermountain Journal of Sciences: Basic Application.*.

[15] Tolcha A., Shibru S., Ayechew B. (2021). Population status and habitat association of Swayne’s hartebeest (*Alcelaphus Buselaphus Swaynei*; Sclater, 1892) in Maze National Park, Southern Ethiopia.

[16] Ford A. T., Goheen J. R., Otieno T. O. (2014). Large carnivores make savanna tree communities less thorny. *Science*.

[17] Crossey B., Chimimba C., Du Plessis C., Ganswindt A., Hall G. (2021). African wild dogs (*Lycaon pictus*) show differences in diet composition across landscape types in Kruger National Park, South Africa. *Journal of Mammalogy*.

[18] Balakrishnan M., Easa P. S. (1986). Habitat preference of large mammals in the Parambikulam Wildlife Sanctuary. Kerala, India. *Biological Conservation*.

[19] Diriba G., Tamene S., Mengesha G., Asefa A. (2020). Diversity of medium and large mammals in the Loka Abaya National Park, Southern Ethiopia. *Ecology and Evolution*.

[20] Girma Z., Worku Z. (2020). Large mammal diversity in Nensebo Forest, Southern Ethiopia. *International Journal of Zoology*.

[21] Stuart C., Stuart T. (2000). *Field Guide to the Large Mammals of Africa*.

[22] Gundogdu E. (2011). Population size, structure and behaviours of wild goat in cehennemdere wildlife improvement area. *Asian Journal of Animal and Veterinary Advances*.

[23] Doku Y., Bekele A., Balakrishnan M. (2006). Human impact on the plains zebra population in Nechisar Plains. Nechi Sar National Park, Ethiopia. *International Journal of Ecology & Environmental Sciences*.

[24] Fetene A., Yeshitela K., Gebremariam E. (2019). The effects of anthropogenic landscape change on the abundance and habitat use of terrestrial large mammals of Nech Sar National Park. *Environmental Systems Research*.

[25] Fetene A., Mengesha G., Bekele T. (2011). Spatial distribution and habitat preferences of selected large mammalian species in the Nech Sar National Park (NSNP), Ethiopia. *Natural Science*.

[26] Girma K., Stellmacher T. (2012). *Contesting the National Park Theorem. Governance and Land Use in Nechsar National Park, Ethiopia*.

